# Genomic Diversity of *Burkholderia pseudomallei* Isolates, Colombia

**DOI:** 10.3201/eid2702.202824

**Published:** 2021-02

**Authors:** Carolina Duarte, Franco Montufar, Jaime Moreno, Dora Sánchez, Jose Yesid Rodríguez, Alfredo G. Torres, Soraya Morales, Adriana Bautista, Mónica G. Huertas, Julia N. Myers, Christopher A. Gulvik, Mindy G. Elrod, David D. Blaney, Jay E. Gee

**Affiliations:** Instituto Nacional de Salud, Bogotá, Colombia (C. Duarte, J. Moreno, D. Sanchez, A. Bautista);; Clínica León XIII Universidad de Antioquia, Medellín, Colombia (F. Montufar);; Centro de Investigaciones Microbiológicas del Cesar, Valledupar, Colombia (J.Y. Rodriguez);; University of Texas Medical Branch, Galveston, Texas, USA (A.G. Torres, J.N. Myers);; Universidad de Santander, Valledupar, Colombia (S. Morales);; Universidad El Bosque, Bogotá, Colombia (M.G. Huertas);; Centers for Disease Control and Prevention, Atlanta, Georgia, USA (C.A. Gulvik, M.G. Elrod, D.D. Blaney, J.E. Gee)

**Keywords:** bacteria, Burkholderia pseudomallei, Colombia, genomic diversity, melioidosis, molecular epidemiology, multilocus sequence typing, phylogeography, whole-genome sequencing

## Abstract

We report an analysis of the genomic diversity of isolates of *Burkholderia pseudomallei*, the cause of melioidosis, recovered in Colombia from routine surveillance during 2016–2017. *B. pseudomallei* appears genetically diverse, suggesting it is well established and has spread across the region.

Melioidosis is caused by the environmental bacterium *Burkholderia pseudomallei.* Infections are acquired by direct contact with the pathogen, most commonly through traumatic inoculation with contaminated soil or water but also by ingestion or inhalation. Symptoms are nonspecific and can include pneumonia, skin lesions, abscess formation, and sepsis (*1*).

In Latin America, melioidosis is believed to be underdiagnosed because of the absence of reliable surveillance and the lack of available diagnostic tools and methods (*2*). Colombia has previously reported cases as sporadic, isolated events in a few geographic areas (*2*,*3*). The aim of this study was to genetically characterize isolates of *B. pseudomallei* recovered from clinical specimens in different departments of Colombia (*4*). (A department in Colombia is a geographic unit composed of municipalities led by a governor.) The goal was to better understand genetic relationships among the isolates from Colombia, as well as their relationships to isolates from other tropical and subtropical regions of the Americas. The study was internally reviewed at the US Centers for Disease Control and Prevention (Atlanta, GA, USA) and determined not to involve human subject research.

Melioidosis is not an officially reportable disease in Colombia, but when cases are identified, department public health laboratories are required to send isolates of *B. pseudomallei* to the Instituto Nacional de Salud. During 2016–2017, a total of 11 isolates of *B. pseudomallei* were recovered from 10 melioidosis patients in the departments of Cesar (n = 4 isolates), Antioquia (n = 4), Casanare (n = 2), and Santander (n = 1) (Appendix). The most common risk factor was diabetes mellitus (n = 6); 4 of the patients died (Table). Cesar, Antioquia, Casanare, and Santander vary in population from a few hundred thousand to >6 million (*4*).

**Table:** Epidemiologic and demographic characteristics of 10 melioidosis patients, Colombia

Isolate	Sequence type	Department	Age, y/sex	Type of sample	Diagnosis	Medical history and risk factors	Outcome
B107	1459	Cesar	71/M	Blood	Sepsis	Arterial hypertension	Died
B108	1459	Cesar	54/M	Right leg injury	Soft tissue infection	Tibial fracture	Recovered
B109	349	Cesar	56/M	Urine	Urinary infection	Diabetes mellitus	Recovered
B197	1463	Cesar	51/F	Bronchoalveolar lavage	Pulmonary melioidosis	Diabetes mellitus, anemic syndrome	Recovered
B198	1701	Casanare	24/M	Blood	Pneumonia	None	Died
B199	518	Casanare	26/M	Blood	Unspecified sepsis	None	Died
B255	92	Santander	68/M	Blood	Sepsis		Recovered
B308*	518	Antioquia	64/M	Tracheal aspirate	Systemic inflammatory response syndrome	Diabetes mellitus	Died
B309*				Blood			
B310	1740	Antioquia	81/F	Tracheal aspirate	Pneumonia	Kidney tumor (in studio), diabetes mellitus, arterial hypertension, hypothyroidism	Recovered
B411	1741	Antioquia	53/F	Blood	Sepsis	Diabetes mellitus	Recovered

*Isolates from the same patient.

We performed whole-genome sequencing of the 11 isolates and deposited sequences at the National Center for Biotechnology Information under BioProject PRJNA638548. Sequences were used for multilocus sequence typing and single-nucleotide polymorphism (SNP) analysis (Appendix[Fig F1]). The multilocus sequence types (ST) we observed were ones previously described, such as ST92, ST349, ST518, and ST1459. Two novel STs from this study were designated ST463 and ST1701. Previous entries in the PubMLST database (http://pubmlst.org) indicate that ST92 has been identified in cases associated with Puerto Rico and Brazil and in 1 person in Switzerland who had travelled to Martinique. ST349 was represented in 2 examples, one from Martinique and the other in a person from Spain who had travelled to West Africa; ST518 is represented in 4 examples. The first was in a person from Arizona, USA, in whom melioidosis developed after sustaining an injury while swimming in Costa Rica (*5*). In addition, ST518 was identified in *B. pseudomallei* isolates from 3 pet green iguanas, 2 of them in California, USA, and 1 in Belgium, all of which were presumably imported from Central or South America (*6*,*7*). ST1459 was noted in 1 isolate from Brazil.

**Figure F1:**
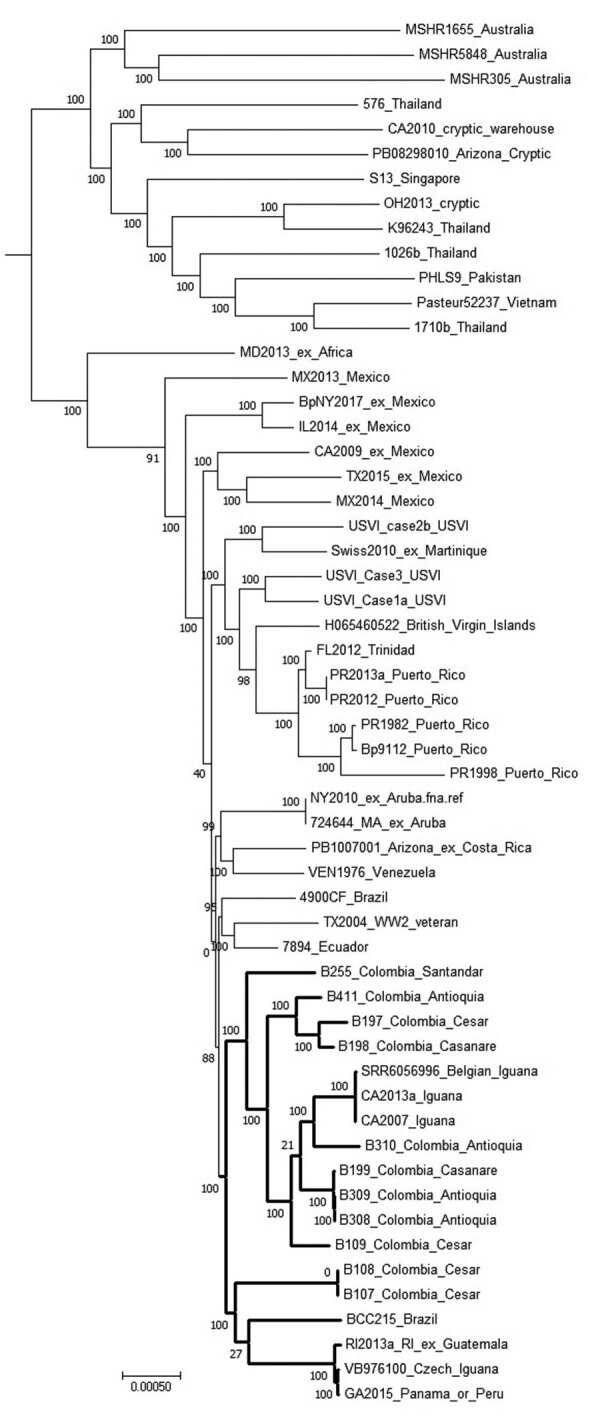
Dendrogram used for characterization of *Burkholderia pseudomallei* genomes from Colombia compared with reference genomes. Tree was generated in MEGA7 software (http://www.megasoftware.net) from results of maximum-parsimony phylogenetic analysis of core single-nucleotide polymorphisms conducted by using Parsnp, a component of the Harvest 1.3 software suite (https://github.com). Bold branches indicate the subclade containing the examples from Colombia along with reference genomes that group with them. Isolates from Colombia also include the department where they originated. Scale bar indicates number of substitutions per single nucleotide polymorphism.

SNP analysis determined from the whole genome sequences indicates that the Colombia isolates (N=11) are within the clade associated with Western Hemisphere *B. pseudomallei* based on a comparison with a panel of reference genomes (N=45) (Figure). Within this clade, a subgroup was resolved containing the Colombia genomes along with ones from Brazil and Guatemala. Also included is a genome from an isolate from a patient who had traveled to both Panama and Peru, as well as isolates from iguanas from California and Belgium, as noted, plus 1 from the Czech Republic that were presumably imported from Central or South America (Figure) (*6**–**8*).

The full panel (N = 56) was also used for quantifying SNP differences among the genomes. Patient isolates B107 and B108 had no SNPs between them, even though they were from different patients, suggesting a common source of infection or a clonal population of *B. pseudomallei* present in different sources. However, isolates B308 and B309 were from the same patient and had 1 SNP between them. The next closest relationship was for B199 (from Casanare), which diverged by 38 SNPs from B308 and by 39 SNPs from B309 (from Antioquia). The phylogenetic SNP tree indicates that isolates from Antioquia, Casanare, and Cesar for the most part do not uniformly group together by department. The largest divergence was seen between B109 and the genomes for B107 and B108, with >6,900 SNPs detected (all from Cesar). The amount of divergence plus the lack of grouping by department, even though we presume that patients’ main exposures would have been within a given department, suggests *B. pseudomallei* is well established in Colombia and has had time to diverge substantially since its introduction. In addition, the genomes from the 2 cases of melioidosis from pet iguanas from California and the 1 from Belgium cluster together with examples from Colombia, suggesting this region or a nearby region may have been the origin of the iguanas. Further studies, especially to recover and test environmental isolates, will improve our understanding of the population structure of *B. pseudomallei* in Colombia and improve the ability of public health stakeholders to respond to cases of melioidosis.

Appendix Additional details on genomic diversity of *Burkholderia pseudomallei* isolates, Colombia.
