# Supplementary material for: Genomic Diversity of Burkholderia pseudomallei Isolates, Colombia
Source: Emerg Infect Dis. 2021 Feb;27(2):655–8. doi: 10.3201/eid2702.202824 (PMC7853579; doi:10.3201/eid2702.202824)
Supplement: Appendix — Additional details on genomic diversity of Burkholderia pseudomallei isolates, Colombia. [file 20-2824-Techapp-s1.pdf]

Article DOI: https://doi.org/10.3201/eid2702.202824

Genomic Diversity of *Burkholderia pseudomallei* Isolates, Colombia

Appendix

Materials and Methods

Through laboratory-based surveillance activities, 11 *Burkholderia pseudomallei* isolates were received by the microbiology group of the Instituto Nacional de Salud in Colombia during 2016–2017. Cultures from blood, sputum, urine, abscesses, and throat swabs generated as part of routine diagnostic procedures were processed according to the protocols of the clinical laboratory of each hospital. We performed preliminary identification of isolates and susceptibility tests using a VITEK 2 (Biomerieux, https://www.biomerieux-usa.com). Isolates that we identified as *Burkholderia* spp., oxidase positive, gram-negative, and non-*Pseudomonas aeruginosa* bacteria, were further tested by MALDI-TOF MS (Bruker, https://www.bruker.com) (*1*).

Six isolates presumptively identified as *B. pseudomallei* or *Burkholderia* spp. were sent to the U.S. Centers for Disease Control and Prevention (CDC) for confirmatory testing, whole genome sequencing, and genetic analysis. DNA from an additional 5 *B. pseudomallei* isolates were also sent to CDC for sequencing and genetic analysis. Colombia has previously reported 20 cases as sporadic, isolated events in a few geographic areas. The departments with melioidosis cases from this study are noted on the map in the Appendix Figure. Accounts of previous cases of melioidosis in Colombia, including maps, have been published elsewhere (*2–10*).

We extracted DNA using the Maxwell RSC Cultured Cells DNA kit on the Promega Maxwell RSC Instrument per the manufacturer’s instructions (https://www.promega.com) or extracted it using a QIAGEN DNeasy Blood & tissue kit (https://www.qiagen.com) from pure overnight culture, according to the manufacturer’s instructions. We quantified DNA concentration and spectrum ratios using a ThermoFisher

Qubit v4.0 fluorometer (https://www.thermofisher.com). We eluted samples in PCR-grade water and RNase A, filtered through a 0.1 µm filter, and checked for sterility before whole genome sequencing (*11*).

We determined isolate sequences from paired-end Illumina reads which were generated on an Illumina MiSeq or iSeq 100 (https://www.illumina.com). We sheared genomic DNA to a mean size of 600 bp using a Covaris LE220 focused ultrasonicator (https://www.covaris.com). We cleaned DNA fragments with a Beckman Coulter Ampure system (https://www.beckmancoulter.com) and used them to prepare dual-indexed sequencing libraries using NEBNext Ultra library prep reagents (New England Biolabs, https://www.neb.com) and barcoding indices synthesized in the CDC Biotechnology Core Facility for the genomes run on the MiSeq. Libraries were analyzed for size and concentration, pooled, and denatured for loading onto the flow cell for cluster generation. We used 2 × 250 bp cycle paired-end sequencing kits to perform sequencing for the Illumina MiSeq. We used a Nextera Flex kit (Illumina) to produce libraries for the iSeq 100 runs, which we performed using 2 × 150 bp cycle paired-end sequencing kits. On completion, sequence reads were filtered for read quality, base called, and demultiplexed using bcl2fastq, version 2.19 (Illumina). We generated assemblies as previously described and assessed them with QUAST v5.0 (https://github.com; *12*,*13*). Features of the genome assemblies are noted in Appendix Table 1.

We submitted genomes to the *B. pseudomallei* MLST website (http://pubmlst.org/bpseudomallei) to identify the sequence types or assign new sequence type identifiers, as needed (*14,15*). We analyzed core SNPs for the genomes from Colombia using Parsnp in the Harvest 1.3 suite (https://github.com) along with a reference panel previously described, plus genomes associated with the Western Hemisphere that have recently become available (*11*,*16*–*19*). The Colombian genomes had an average of 3,822 SNPs in nonprotein-encoding (intergenic) positions compared with K96243; 2.1 × more SNPs were observed in genes that had no predicted amino acid changes (Appendix Table 2). The dendrogram was generated in MEGA 7 (https://www.megasoftware.net) (*20*). SNP effects of the Colombian isolates compared with the K96243 reference strain were predicted with SnpEff v4.3t (https://github.com; *21*).

References

1. Inglis TJ, Rolim DB, Rodriguez JL. Clinical guideline for diagnosis and management of melioidosis. Rev Inst Med Trop São Paulo. 2006;48:1–4. PubMed https://doi.org/10.1590/S0036-46652006000100001

2. Montúfar FE, Ochoa JE, Ortega H, Franco L, Montúfar MC, Monsalve A, et al. Melioidosis in Antioquia, Colombia: an emerging or endemic disease? A cases series. Int J Infect Dis. 2015;37:50–7. https://doi.org/10.1016/j.ijid.2015.05.023

3. Severiche D. Community-acquired pneumonia due to *Pseudomonas pseudomallei* [in Spanish]. Rev Colomb Neumol. 1998;10:188–92.

4. Gonzalez G, Mantilla WA, Rada R. *Burkholderia pseudomallei* pneumonia and osteomyelitis, report of a clinical case [in Spanish]. Rev Med (São Paulo). 2009;17:146–9.

5. Rodriguez JY, Morales-Lopez SE, Rodriguez GJ, Alvarez-Moreno CA, Esquea K, Pinzon H, et al. Case series study of melioidosis, Colombia. Emerg Infect Dis. 2019;25:1534. PubMed https://doi.org/10.3201/eid2508.170786

6. Guzman-Gomez L, Agudo Bilbao M, Peiro-Callizo E, Salas C. Melioidosis imported from Colombia to Spain [in Spanish]. Enferm Infecc Microbiol Clin. 2015;33:214–6. PubMed https://doi.org/10.1016/j.eimc.2014.06.003

7. Osorio Carmona HE, Duran LF. Melioidosis: Report of a case in the department of Huila—Colombia. RFS Revista Facultad de Salud. 2014;6:56–9. https://doi.org/10.25054/rfs.v6i2.166

8. Rodriguez JY, Alvarez-Moreno CA, Cortes JA, Rodriguez GJ, Esquea K, Pinzon H, et al. Melioidosis in Colombia, description of a clinical case and epidemiological considerations. Biomedica. 2019;39:10–8. PubMed https://doi.org/10.7705/biomedica.v39i3.4534

9. Rosero Arellano CH, Gomez G, Gutierrez Sanjuan OI. Six-year history of cough and dyspnea in a young adult with chronic pulmonary melioidosis. A case report [in Spanish]. Medicina & Laboratorio. 2013;19:465–72.

10. Camargo N, Casadiego GK, Fernandez DA, Millan LV, Hernandez AK, Vargas S, et al. A young diabetic patient with sepsis after gardening. Open Forum Infect Dis. 2020;7:ofaa159. PubMed https://doi.org/10.1093/ofid/ofaa159

11. Gee JE, Gulvik CA, Elrod MG, Batra D, Rowe LA, Sheth M, et al. Phylogeography of *Burkholderia pseudomallei* isolates, Western Hemisphere. Emerg Infect Dis. 2017;23:1133–8. PubMed https://doi.org/10.3201/eid2307.161978

12. Nouioui I, Klenk HP, Igual JM, Gulvik CA, Lasker BA, McQuiston JR. *Streptacidiphilus bronchialis* sp. nov., a ciprofloxacin-resistant bacterium from a human clinical specimen; reclassification of *Streptomyces griseoplanus* as *Streptacidiphilus griseoplanus* comb. nov. and emended description of the genus *Streptacidiphilus*. Int J Syst Evol Microbiol. 2019;69:1047–56. PubMed https://doi.org/10.1099/ijsem.0.003267

13. Gurevich A, Saveliev V, Vyahhi N, Tesler G. QUAST: quality assessment tool for genome assemblies. Bioinformatics. 2013;29:1072–5. PubMed https://doi.org/10.1093/bioinformatics/btt086

14. Jolley KA, Maiden MC. BIGSdb: Scalable analysis of bacterial genome variation at the population level. BMC Bioinformatics. 2010;11:595. PubMed https://doi.org/10.1186/1471-2105-11-595

15. Godoy D, Randle G, Simpson AJ, Aanensen DM, Pitt TL, Kinoshita R, et al. Multilocus sequence typing and evolutionary relationships among the causative agents of melioidosis and glanders, *Burkholderia pseudomallei* and *Burkholderia mallei*. J Clin Microbiol. 2003;41:2068–79. PubMed https://doi.org/10.1128/JCM.41.5.2068-2079.2003

16. Treangen TJ, Ondov BD, Koren S, Phillippy AM. The Harvest suite for rapid core-genome alignment and visualization of thousands of intraspecific microbial genomes. Genome Biol. 2014;15:524. PubMed https://doi.org/10.1186/s13059-014-0524-x

17. Elschner MC, Hnizdo J, Stamm I, El-Adawy H, Mertens K, Melzer F. Isolation of the highly pathogenic and zoonotic agent *Burkholderia pseudomallei* from a pet green iguana in Prague, Czech Republic. BMC Vet Res. 2014;10:283. PubMed https://doi.org/10.1186/s12917-014-0283-7

18. Hellebuyck T, Wattiau P, Boyen F, Moeremans I, Roosens NH, Vanneste K, et al. Isolation of *Burkholderia pseudomallei* from a pet green iguana, Belgium. Emerg Infect Dis. 2018;24:2331–3. PubMed https://doi.org/10.3201/eid2412.171661

19. Donahue MA, Newcomb G, Spinella S, Prasad P, Liesveld J, Egan CT, et al. Melioidosis in a traveler returning from Cabo, Mexico. Open Forum Infect Dis. 2019;6:ofz005. PubMed https://doi.org/10.1093/ofid/ofz005

20. Kumar S, Stecher G, Tamura K. MEGA7: Molecular Evolutionary Genetics Analysis version 7.0 for bigger datasets. Mol Biol Evol. 2016;33:1870–4. PubMed https://doi.org/10.1093/molbev/msw054

21. Cingolani P, Platts A, Wang LL, Coon M, Nguyen T, Wang L, et al. A program for annotating and predicting the effects of single nucleotide polymorphisms, SnpEff: SNPs in the genome of *Drosophila melanogaster* strain w1118; iso-2; iso-3. Fly (Austin). 2012;6:80–92. PubMed https://doi.org/10.4161/fly.19695

Appendix Table 1. General features of Colombian genome assemblies.

Sample	Contigs*	Total length (bp)	Largest contig, bp	GC, %†	N50‡	L50§
B107	289	7,125,249	137,454	68.08	43,818	53
B108	463	7,106,012	99,738	68.04	28,313	76
B109	444	7,134,078	134,685	68.06	31,337	68
B196	585	7,226,750	108,429	67.76	24,102	87
B197	466	7,204,391	117,090	68.01	28,036	78
B198	394	7,008,319	120,168	68.22	34,483	62
B199	259	7,040,139	292,250	68.25	51,517	39
B255	536	7,204,800	98,505	67.97	27,345	81
B308	311	7,016,254	170,164	68.24	44,603	49
B309	296	7,018,475	195,367	68.25	48,879	44
B310	321	7,086,990	152,984	68.14	40,384	52
B411	357	7,026,297	126,891	68.19	40,276	55

*No. of contiguous sequences assembled from short raw Illumina sequences
†Percentage of a genome assembly containing Guanine and Cytosine nucleotides
‡Length of the smallest contig, which together with larger contigs comprise half of the total assembly size
§Smallest contig quantity to make up 50% of the total assembly size

Appendix Table 2. Predicted mutation consequences of SNPs observed in the Colombian isolates compared with the reference strain K96243 (GCA 000959285.1).

Sample	Synonymous	Missense	Intergenic	Noncanonical start codon	Start codon lost	Stop codon gained	Stop codon lost
B107	7920	9400	3799	15	33	307	0
B108	7957	9386	3872	15	29	302	0
B109	7935	9307	3807	12	31	307	0
B196	7982	9445	3898	14	35	319	0
B197	7929	9415	3759	15	32	289	0
B198	7993	9376	3860	16	29	312	0
B199	7872	9271	3687	12	33	314	0
B255	7924	9387	3796	15	33	307	0
B308	7908	9373	3833	15	33	306	0
B309	7990	9386	3873	13	30	310	0
B310	7920	9400	3801	15	33	307	0
B411	7984	9518	3873	13	30	310	0

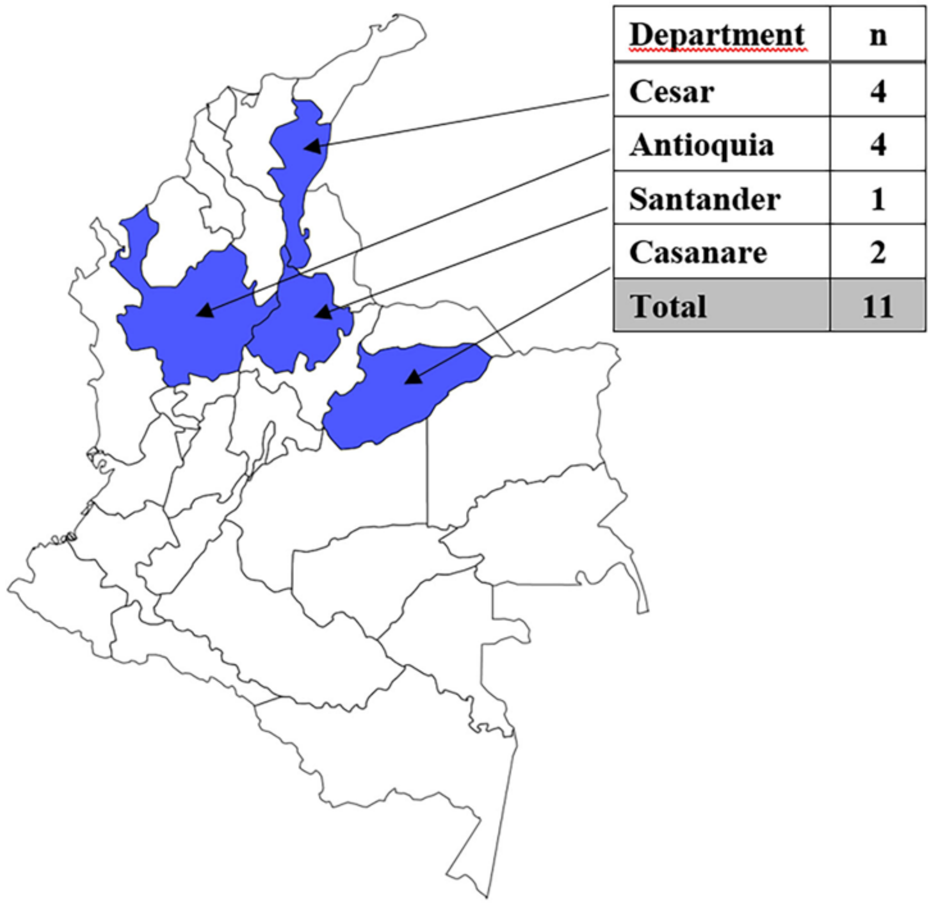

Appendix Figure. Map of Colombia showing number of melioidosis cases by department.
